# Eruptions Caused by Balsam Copavia

**Published:** 1828-04

**Authors:** T. Hewson


6. *Eruptions caused by Balsam Copavia.* In the 9th number of the North American Medical and Surgical Journal, Dr. Hewson, one of the most estimable members of the profession, in Philadelphia, has published a short account of four cases of eruptive disease, apparently caused by the use of balsam copaiva. Two of them were distinctly marked, as Urticaria or Nettle-rash; the others bore more resemblance to Roseola. In all, the efflorescence disappeared, upon laying aside the balsam, and administering a solution of super tartrate of potash, with a vegetable diet.

It is, perhaps, not undeserving of remark, that the whole of these patients laboured under mucous discharges—the first had fluor albus, the others gonorrhœa. Now, as papular eruptions, according to Carmichael, frequently occur in the latter malady, may it not be possible, that the cutaneous affections of Dr. Hewson's patients was, some way or other, connected with the diseases for which the balsam was administered?

